# Controlling hypertension immediately post stroke: a cost utility analysis of a pilot randomised controlled trial

**DOI:** 10.1186/1478-7547-8-3

**Published:** 2010-03-23

**Authors:** Edward CF Wilson, Gary A Ford, Tom Robinson, Amit Mistri, Carol Jagger, John F Potter

**Affiliations:** 1 Faculty of Health, University of East Anglia, Norwich, UK; 2 Stroke Research Group, Institute for Ageing and Health, Newcastle University, UK; 3 Ageing & Stroke Medicine, Department of Cardiovascular Sciences, University of Leicester, Leicester General Hospital, Leicester, UK; 4 Institute for Ageing and Health, Newcastle University, Newcastle Upon Tyne, UK

## Abstract

**Background:**

Elevated blood pressure (BP) levels are common following acute stroke. However, there is considerable uncertainty if and when antihypertensive therapy should be initiated.

**Method:**

Economic evaluation alongside a double-blind randomised placebo-controlled trial (National Research Register Trial Number N0484128008) of 112 hypertensive patients receiving an antihypertensive regimen (labetalol or lisinopril) within 36 hours post stroke versus 59 receiving placebo. Outcomes were incremental cost per incremental: QALY, survivor, and patient free from death or severe disability (modified Rankin scale score < 4) at three months and 14 days post stroke.

**Results:**

Actively treated patients on average had superior outcomes and lower costs than controls at three months. From the perspective of the acute hospital setting, there was a 96.5% probability that the incremental cost per QALY gained at three months is below £30,000, although the probability may be overstated due to data limitations.

**Conclusion:**

Antihypertensive therapy when indicated immediately post stroke may be cost-effective compared with placebo from the acute hospital perspective. Further research is required to confirm both efficacy and cost-effectiveness and establish whether benefits are maintained over a longer time horizon.

## Background

Approximately 52,000 patients experience first stroke [1], and 135,000 experience first or recurrent stroke in England and Wales each year [2]. It is the third biggest cause of death and the most important single cause of severe adult disability [3]. The societal cost of stroke to England and Wales is estimated at £7bn, of which 40% are direct care costs, 35% informal care, and the remaining 25% indirect costs (lost productivity) [4].

Elevated blood pressure (BP) levels are common following onset of acute stroke, and observational data suggest that both high and low BP levels are associated with poor short and long term prognosis [5-16]. The acute management of post-stroke BP changes is a matter of some debate, with considerable differences of opinion on when to initiate antihypertensive therapy [17]. A Cochrane review of BP manipulation following stroke concluded that there was insufficient evidence to evaluate the effect of changes on patient outcomes [18].

In view of the uncertainty surrounding appropriate response to BP control in the acute post-stroke phase, the Control of Hypertension and Hypotension Immediately Post Stroke (CHHIPS) trial (National Research Register Trial Number N0484128008) aimed to establish the safety, efficacy and cost-effectiveness of reducing BP with labetalol or lisinopril in hypertensive patients with acute cerebral infarction or haemorrhage, and of raising BP with phenylephrine in hypotensive patients with ischaemic stroke.

As resources are finite, decision making requires consideration not only of the benefits to a patient of a health care intervention, but its impact on other patients consuming other diverse health care services: committing resources to one intervention means they cannot be employed, or must be withdrawn from, elsewhere. An economic evaluation considers the cost and consequences of two or more treatment strategies, and shows the change in both cost and outcome by adopting a new strategy in place of old [19]. The change in cost divided by the change in outcome (the incremental cost-effectiveness ratio or ICER) is then compared with a maximum 'threshold'. This threshold can be interpreted as the cost-effectiveness of the least efficient service currently provided by the health service (although alternative interpretations of the threshold exist). If the ICER is below this threshold, adopting the new treatment (and by implication ceasing the least efficient service) will improve the net health gain to the population. Conversely, adopting a treatment whose ICER is above the threshold will lead to a net reduction in health gain to the population. An outcome measure commonly used to make these comparisons is the Quality Adjusted Life Year (QALY), and the threshold in the UK is considered to be in the region of £20,000 - £30,000 per QALY gained [20].

We report a cost-utility and cost-effectiveness analysis of therapeutically reducing blood pressure compared with no therapeutic reduction in blood pressure in hospitalised hypertensive patients with acute cerebral infarction or haemorrhage.

## Methods

Full details of the methods and outcome measures in the study are reported elsewhere [21-23]. The study was designed to include both pressor and depressor trial arms. Due to low recruitment, the pressor arm of the trial was terminated early. We therefore report costs and outcomes relating to the depressor arm only.

Briefly, 179 patients aged 18+ years with a clinical diagnosis of stroke (cerebral infarct or haemorrhage) with onset ≤ 36 hours and systolic blood pressure (SBP) ≥ 160 mmHg were enrolled into this randomised double-blind placebo-controlled trial. Exclusion criteria included on antihypertensive therapy at time of stroke onset (amended during study to allow inclusion of dysphagic patients on antihypertensive therapy) or an urgent indication for BP lowering, significant co-morbidity, or a life expectancy ≤ six months due to non-stroke causes prior to stroke onset.

Following baseline assessment (SBP levels, time of stroke onset, swallowing status, functional assessments including modified Rankin scale (mRS) and National Institute of Health Stroke Scale (NIHSS)), patients were randomised on a 2:1 ratio between active treatment and placebo.

Active treatment comprised stepped doses of oral (for non-dysphagic) or intravenous/sublingual routes of labetalol or lisinopril respectively with a target SBP of 145-155 mmHg or a SBP fall of ≥ 15 mmHg. Additional doses were administered at 4 and 8 hours post randomisation if targets were not met. Controls were administered matching placebo, and the regimen continued for 14 days post randomisation. Dysphagic patients underwent similar titrated dosing but with sublingual lisinopril 5 mg, intravenous labetalol 50 mg or matching placebo for 72 hours, then oral therapy (if possible), or via nasogastric tube until day 14. Subsequently all patients followed local guidelines as regards antihypertensive therapy (usually an ACE inhibitor and/or diuretic). At day 14 and 3 months post randomisation, mRS was completed.

Baseline and two week assessments were performed by research staff at the local centres. Three month follow-up was by telephone administered from the trial coordinating centre. Where participants were not able to recall date of discharge at the three month follow-up, the local research staff were contacted to obtain the date from hospital records.

The primary outcomes were incremental cost per incremental survivor and incremental cost per incremental QALY gained at 3 months post randomisation with active treatment versus placebo. Secondary analyses comprised incremental cost per incremental: patient with death or severe disability (defined as mRS score < 4) at 14 days and 3 months, and survivor and QALY gained at 14 days.

Utilities were mapped to mRS scores estimated from a study of 459 individuals eliciting utilities from mRS scores using the time trade-off (TTO) approach [24].

The analysis was conducted from the perspective of the acute hospital. Hence resource use data comprised patient length of stay and study drug consumption. The price year of the study was 2006. Length of stay (LoS) was calculated as the difference between date of death or discharge and date of randomisation. The bulk of hospitalisation costs tend to be skewed towards the first few days of admission and the National Schedule of Reference Costs 2006 [25] estimates the mean cost of a stroke admission at £2642, with a mean length of stay of 11 days, and a daily cost of excess bed-days of £176. We therefore approximated the cost of an admission as:

Per patient cost of study drugs was estimated as number of tablets or vials multiplied by unit cost (lisinopril @ £1.34/28 5 mg tabs, labetalol @ £3.79/56 50 mg tabs and £2.12/20 ml ampoule[26]). Placebo was costed at zero.

We present results as quantities of resource use and total cost, and outcomes by treatment group (active treatment vs placebo). The incremental cost-effectiveness ratio (ICER) was calculated as

Uncertainty in the point estimate ICER was investigated by means of a non-parametric bootstrap with 1000 replications. This was used to estimate confidence intervals around incremental cost and outcomes, and to generate the cost-effectiveness acceptability curve (CEAC). The CEAC shows the treatment (active or placebo) with the highest probability of being cost-effective at varying thresholds of willingness to pay for a unit of outcome, and is a means of expressing uncertainty around point estimates [27].

**Figure 1:**
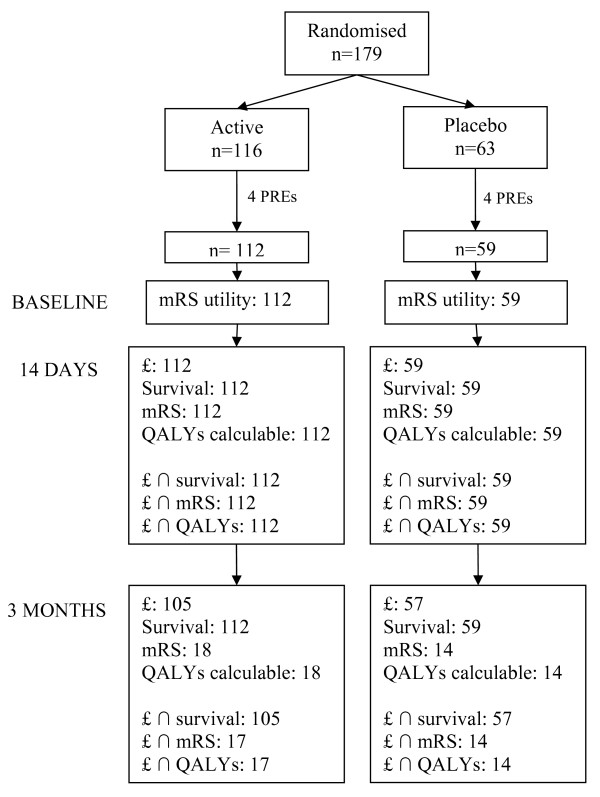
**Complete case analysis sample sizes**.

Results are presented as cost of each arm and increment, outcome from each arm and increment, and incremental cost-effectiveness (Table 1). The figures reported in Table 1 are based on complete case analysis (observations for which both cost and outcome data were available). Tables 2 and 3 report disaggregated resource use and cost, and outcomes using all observations for which cost or outcomes data were available (see Figure 1 for details).

**Table 1:** Cost utility and cost effectiveness analyses at 14 days and 3 months (Complete case analysis)

	n		£			Outcome				
	A	P	A	P	Increment (95% CI)	A	P	Increment (95% CI)	ICER	P(ICER ≤ £30k)**
1. 14d survival*	112	59	2553	2525	28 (-228, 269) §	0.955	0.898	0.057 (-0.028, 0.144)	£490	
2. 14d D&D†	112	59	2553	2525	28 (-215, 278) §	0.393	0.407	-0.014 (-0.169, 0.149)	[P dominant]	
3. 14d CUA‡	112	59	2553	2525	28 (-226, 268) §	0.028	0.027	0 (-0.001, 0.002)	£76,162	45.9%
4. 3 m survival*	105	57	8234	9233	-1000 (-3760, 1588)	0.905	0.789	0.115 (0.001, 0.232)	[A dominant]	
5. 3 m D&D†	17	14	5324	10835	-5511 (-15183, 1221)	0.412	0.071	0.340 (0.080, 0.588)	[A dominant]	
6. 3 m CUA‡	17	14	5324	10835	-5511 (-15712, 1311)	0.098	0.054	0.044 (0.000, 0.086)	[A dominant]	96.5%

* Outcome = proportion surviving; † Outcome = proportion not dead or dependent (defined as mRS<4). ‡ Outcome = QALYs gained; §Differences in 95%CI around incremental cost in analyses 1, 3 & 5 due to random error from non-parametric bootstrap.

** Threshold of £30,000 only appropriate to £/QALY.

**Table 2:** Mean Resource use and cost at 14 days and 3 months

	14 days					3 months				
	N					N				
	A	P	A	P	A-P	A	P	A	P	A-P
Mean (SE) Los (days)	**112**	**59**	**11.49 (0.402)**	**11.36 (0.577)**	**0.14**	105	57	43.77 (3.38)	49.47 (7.28)	-5.7
Median (IQR) LoS (days)	**112**	**59**	**14 (9, 14)**	**14 (10,14)**	**0**	105	57	38 (7,84)	34 (10,84)	4.0
Patients still hospitalised n (%)	**112**	**59**	**76 (67.9)**	**38 (64.4)**	**3.45%**	105	57	29 (27.6)	16 (28.1)	-0.45%
Study drug consumption, vials. Mean (SE)	**112**	**59**	**4.7 (0.7)**	**5.7 (1.1)**	**-1.02**	112	59	4.7 (0.7)	5.7 (1.1)	-1
Study drug consumption, tabs. Mean (SE)	**112**	**59**	**32.53 (2.3)**	**45.68 (3.9)**	**-13.15**	112	59	32.5 (2.3)	45.7 (3.9)	-13.15
Cost of hospitalisation, £, mean (SE)	**112**	**59**	**2,548 (71)**	**2,525 (101)**	**23.78**	105	57	8,230 (594)	9,233 (1282)	-1,003.60
Cost of study drugs, £, mean (SE)	**112**	**59**	**4 (1)**	**0 (0)**	**4.14**	105	59	4 (1)	0 (0)	4
Total cost, £, mean (SE)	**112**	**59**	**2,553 (71)**	**2,525 (101)**	**27.93 (124)**	105	57	8,234 (594)	9,233 (1282)	-999.50 (1413)

SE = Standard error of the mean, IQR = Inter-quartile range, A = active (labetalol or lisinopril), P = placebo. Note figures may vary

from those reported in Table 1 due to numbers of observations included (see Figure 1)

**Table 3:** Outcomes at 14 days and 3 months

	N					
	**A**	**P**	**A**	**P**	**A-P**	**P-value**

**Mean (SE) utility**						

Baseline	112	59	0.892 (0.007)	0.899 (0.008)	-0.007	

14 days	112	59	0.551 (0.022)	0.526 (0.035)	0.026	0.519

3 months	18	14	0.366 (0.100)	0.088 (0.060)	0.278	0.035

**Mean (SE) QALYs gained**						

14 days	112	59	0.028 (0.0005)	0.027 (0.0007)	0.000	0.650

3 months	18	14	0.102 (0.0185)	0.054 (0.0116)	0.048	0.051

**Survival n (%)**						

14 days	112	59	107 (95.54)	53 (89.83)	5.71%	0.148

3 months	112	59	102 (91.07)	47 (79.66)	11.41%	0.034

**mRS<4 n (%)**						

Baseline	112	59	112 (100)	59 (100)	0.00%	

14 days	112	59	44 (39.29)	24 (40.68)	-1.39%	0.860

3 months	18	14	8 (44.44)	1 (7.14)	37.30%	0.020

*Based on mapped mRS scores

**t-test for continuous variables, χ^2 ^for proportions

A = active, P = placebo. Note figures vary from those reported in Table 1 due to numbers of observations included (see Figure 1).

## Results

Of 179 patients randomised to the trial, eight were withdrawn post randomisation (see Potter et al. [23] for details of post-randomisation exclusions). Resource use data at 14 days and three months were available on 171 (Active = 112, Placebo = 59) and 162 (Active = 105, Placebo = 57) patients respectively. Utility data based on mRS score at baseline and 14 days were available on all 171 patients. However at three months, mRS and hence mRS-based utilities and QALYs gained were available on 32 (Active = 18, Placebo = 14) patients. Survival status up to three months was recorded in all 171 patients. Therefore full cost and outcomes data were available on 171 (Active = 112, Placebo = 59) patients at 14 days. At three months cost and survival data were available on 162 (Active = 105, Placebo = 57) patients, and cost and death/disability and cost and QALY data on 31 (Active = 17, Placebo = 14) patients (Figure 1).

There were no substantial differences in baseline characteristics between active and placebo treatment groups [23].

### Cost effectiveness

There were no significant differences in cost or outcomes at 14 days (Table 1, analyses 1-3). At three months, active treatment per patient was (non-significantly) decreased by between £1000 and £5511 (Analyses 4-6 Table 1), with a gain of 0.044 QALYs (95% CI 0.000, 0.086; Analysis 6 Table 1). Survival at three months favoured active treatment (+11.5%, 95%CI: +0.1%, +23.2%; Analysis 4 Table 1), as did proportion free from death or severe disability (+34.0%, 95%CI: +8.0%, +58.8%; Analysis 5 Table 1). The difference in the estimated cost increment between analysis 4 and analyses 5 and 6 is due to missing data: the figure quoted in analysis 4 (£1000) is based on substantially more observations than that in analyses 5 and 6 (£5511), and is therefore subject to less sampling uncertainty.

At three months, therefore, according to all outcome measures, active treatment 'dominates' placebo (it is on average less expensive and more effective). We estimate a 96.5% probability of the incremental cost per QALY gained being below £30,000 (Table 1 Analysis 6), indeed irrespective of the threshold, the probability that treatment is cost-effective never falls below 92%.

The above figures are based on complete case analysis. That is, observations were included in analyses 1-6 only where complete cost and outcome data were available (see Figure 1). We had complete survival data on all 171 patients at three months. However, we were only able to measure mRS and hence QALYs gained on 32 patients at 3 months. Therefore the estimate of incremental cost reported above does not include all observations for which cost data were available. Looking just at resource use data (and hence based on n = 105 active + 57 placebo), we estimate an incremental cost at 3 months of -£1000 (95% CI: -3450, 1451; Table 2). Similarly, we estimate incremental QALYs at 3 months at +0.048 (-0.0002, 0.0956; Table 3).

## Discussion

To our knowledge, this is the first study examining the cost-effectiveness of antihypertensive medication immediately post stroke. Other studies have been in the context of primary or secondary prevention of cardio- or cerebrovascular events in hypertensive patients. These studies largely favour the use of preventative pharmacotherapy [28-30].

On average over three months, we found active treatment within the first 2 weeks of stroke onset to be both cost saving and outcome improving, leading to active treatment dominating placebo. However there are important caveats to bear in mind in interpreting the results. It should be noted that 95% confidence intervals around increments were of borderline statistical significance (e.g. Table 1, outcomes analyses 4, 5 and 6). It is highly likely that the analyses with small sample sizes (e.g. 5 and 6) are subject to selection bias due to potential correlation between health status and probability of providing outcomes data at three months (this is likely 'U-shaped': sicker individuals are less likely to respond to request for longer term follow-up data, whilst death is relatively easy to establish. Indeed, we had mRS and QALY data on 23 (11, 12) of 31 patients by virtue of knowledge of date of death).

This was a trial for which data collection proved to be problematic, particularly in terms of disability status at three month follow-up. The primary objective of the study was to assess whether disability and death at two weeks post stroke was affected by drug induced reduction of BP [23]. Study recruitment was only 11% of that for which it was powered, for a variety of reasons including the inherent difficulty in recruiting patients within the allowed time frame *post ictus*, and higher than anticipated prevalence of pre-treated hypertension (one of the exclusion criteria).

The economic evaluation component of this study was added following commencement of the trial via a protocol amendment, with research resources permitting only limited data collection. Therefore the analysis relied almost exclusively on patient-reported length of stay to determine the cost of active and placebo treatments (the cost of the study drugs was trivial), and the perspective of the analysis was thus restricted to the acute hospital admitting the stroke patient.

The use of self-reported length of stay is a common method for data collection in economic evaluations alongside trials. However, this is subject to recall bias. Studies of the reliability of self-reported data have reported mixed results [31,32]. The impact of this on the study depends on whether the average errors in length of stay are equal between the arms. Randomisation should ensure an even distribution of patients more or less likely to misreport their length of stay ceteris paribus, but it is likely the error will increase with increasing length of stay. In common with all studies collecting resource use data in this way, this must be borne in mind in interpreting the results.

Costing based on length of stay with drug costs added to this may risk double counting if the unit cost used factors in an allowance for drugs. This is an issue common to many economic evaluations, and care must be taken to be sure of what is included in 'per episode' unit costs. In the context of this study, as drug costs were such a trivial component, the impact on the results would be negligible.

We did not document readmissions within this study. However, for this to affect the conclusion of the study, we estimate that patients in the treatment arm would on average, need 2.3 to 2.5 additional readmissions per patient over the three months compared with placebo. We consider such a large difference to be unlikely, indeed a priori it may be expected for there to be fewer readmissions in the active treatment arm. (Please see Appendix 1 for details).

The EQ-5D generic quality of life instrument was included within this study by protocol amendment. As this was after baseline measurements had been taken, and due to the small numbers of observations, it was decided to map the mRS scores to utilities and hence QALYs gained, rather than use the EQ-5D data [23]. The analysis did not take into account uncertainty in the TTO valuations of the MRS scale [24]. Therefore we may have underestimated the decision uncertainty, although this would not affect the point estimate results.

We only had relatively small numbers of observations for analyses 5 and 6 (reporting incremental cost per incremental death and disability avoided and QALY; Table 1). There is therefore danger of the groups being unbalanced. A comparison of baseline characteristics of patients included in these analyses shows that they remain broadly balanced (the tables in additional files 1 and 2 show the baseline characteristics of patients included in analysis 4 and analyses 5 & 6 respectively), and results of these analyses are consistent with those of analysis 4, based on a much larger patient sample.

Given the limitations outlined above, the question that must be asked is whether any conclusions can be drawn from such data about a) cost-effectiveness from the acute setting perspective, and b) the generalisability of this restricted analytic perspective to wider societal cost-effectiveness over a longer horizon. Length of stay has been shown to be the major determinant of acute care cost [33,34] and therefore our cost estimates could be plausible indicators of the incremental cost of treating patients under active or placebo treatment in the acute setting. The issue of generalisability to wider perspectives is of particular relevance given the high care needs and associated cost of many stroke survivors (both in terms of health and social services, and informal carer time [4,35]).

This can only by answered either through long-term prospective studies, or through decision analytic modelling. Such a prospective study may be prohibitively expensive and time consuming to conduct. The modelling approach is therefore recommended as a means of generating an answer within a reasonable time frame, and the results of this study should be seen as a valuable input into such an exercise, rather than a definitive estimate of the cost-effectiveness of antihypertensive medication immediately post stroke. Once such a model has been developed, value of information analysis may be used to estimate the likely return from a larger scale (and longer term) trial [36].

Future trials of treatments in this area wishing to incorporate an economic aspect to their investigations should include a) generic quality of life measurement alongside any disease specific or clinical endpoints and b) resource use data collection from the outset. Consideration should be given as to whether at the very least quality of life and place of residence (i.e. own home, care home, nursing home) could be relatively easily measured at, say, six months and one year post intervention to lengthen the time horizon of any such study at minimal additional research cost.

## Conclusion

Antihypertensive therapy in hypertensive patients immediately post stroke may be effective and cost-effective compared with placebo from the acute hospital perspective at three months *post ictus*. Further research, in particular decision analytic modelling, is required to confirm both efficacy and cost-effectiveness and whether benefits are maintained over a longer time horizon. The data from this study form a useful input into such a model.

## Competing interests

The authors declare that they have no competing interests.

## Authors' contributions

JFP was the principal investigator, developed the trial, sought and obtained funding. CJ oversaw the statistical analysis. AKM was the CHHIPS trial coordinator and responsible for data management. TGR & GAF were co-investigators responsible for developing the trial, applying for trial funding and were members of the trial steering committee. EW carried out the economic evaluation and drafted the manuscript. All authors read and reviewed manuscript drafts, and approved the final version.

## Appendix 1: The estimated impact of excluding readmissions

• At three months, point estimate results were that intervention was £5,324 less expensive than control, and resulted in 0.044 more QALYs, yielding an ICER of -£121,000 (intervention dominant).

• For the ICER to be below £20,000, the cost in the intervention arm could rise by £6204 (yielding an incremental cost of +£880 as £880/0.044 = £20,000).

• The mean cost of a stroke admission in the study price year of 2006 was £2642. Therefore the intervention is still cost-effective compared with control so long as there were less than 6204/2642 = 2.3 more admissions per patient, on average, in the intervention arm compared with control over the three month period. (Note this is not total admissions, but 2.3 additional admissions compared with the control arm.)

• for the ICER to be below £30,000, intervention arm patients must have no more than a average of 2.5 admissions per patient over the three month period.

## Supplementary Material

Additional file 1 **Table A2.1**. Baseline characteristics of patients included in analysis 4. Click here for file

Additional file 2 **Table A2.2**. Baseline characteristics of patients included in analyses 5 and 6. Click here for file
