# Supplementary material for: Controlling hypertension immediately post stroke: a cost utility analysis of a pilot randomised controlled trial
Source: Cost Eff Resour Alloc. 2010 Mar 23;8:3. doi: 10.1186/1478-7547-8-3 (PMC2853505; doi:10.1186/1478-7547-8-3)
Supplement: Additional file 1 — Table A2.1. Baseline characteristics of patients included in analysis 4. [file 1478-7547-8-3-S1.DOC]

Table A2.1 Baseline characteristics of patients included in analysis 4

|  | Active  n=105 | Placebo  n=57 |
| --- | --- | --- |
| Male Gender, n (%) | 61 (58) | 30 (53) |
| Age (years) | 74 (11) | 74 (11) |
| SBP (mmHg) | 182 (17) | 181 (16) |
| DBP (mmHg) | 95 (13) | 96 (12) |
| OCSP (%)  Total  Partial  Lacunar  Posterior  Unknown | 36 (34)  33 (31)  26 (25)  9 (9)  1 (1) | 21 (37)  17 (30)  16 (28)  3 (5)  0 (0) |
| mRS Score, n (%)  0  1  2  3 | 77 (73)  16 (15)  7 (7)  5 (5) | 42 (74)  9 (16)  5 (9)  1 (2) |
| NIHSS, median (IQR) | 9 (5-16) | 9 (4-17.5) |
| Dysphagic, n (%) | 50 (48) | 26 (46) |
| No history of stroke, n (%) | 96 (95) | 54 (91) |
| No history of TIA, n (%) | 96 (91) | 53 (93) |
| No Diabetes, n (%) | 98 (93) | 53 (93) |
| Smoking, n (%)  No  Ex-smoker  Current smoker | 53 (51)  33 (31)  19 (18) | 25 (44)  17 (30)  15 (26) |
| No hypercholesterolaemia, n (%) | 76 (72) | 35 (61) |
| No history of IHD, n (%) | 92 (88) | 52 (91) |
| Type of stroke, n (%)  Ischaemic  PICH  No relevant abnormality on scan  Died before scan | 62  17  26  0 | 34  7  14  2 |
