# Supplementary material for: Controlling hypertension immediately post stroke: a cost utility analysis of a pilot randomised controlled trial
Source: Cost Eff Resour Alloc. 2010 Mar 23;8:3. doi: 10.1186/1478-7547-8-3 (PMC2853505; doi:10.1186/1478-7547-8-3)
Supplement: Additional file 2 — Table A2.2. Baseline characteristics of patients included in analyses 5 and 6. [file 1478-7547-8-3-S2.DOC]

Table A2.2 Baseline characteristics of patients included in analyses 5 and 6

|  | Active  n=17 | Placebo  n=14 |
| --- | --- | --- |
| Male Gender, n (%) | 11 (65) | 6 (42.9) |
| Age, years (SE) | 77 (1.1) | 79 (1.4) |
| SBP, mmHg (SE) | 175 (16.5) | 184 (15.6) |
| DBP, mmHg (SE) | 90 (13.5) | 97 (12.1) |
| OCSP, n (%)  Total  Partial  Lacunar  Posterior  Unknown | 9 (53)  2 (12)  5 (29)  1 (6)  0 | 10 (71)  3 (21)  0  1 (7)  0 |
| mRS Score, n (%)  0  1  2  3 | 13 (77)  1 (6)  0  3 (18) | 10 (71)  3 (21)  0  1 (8) |
| NIHSS, median (IQR) | 21 (12 - 24) | 15 (6 - 22) |
| Dysphagic, n (%) | 11 (65) | 12 (86) |
| No history of stroke, n (%) | 17 (100) | 13 (93) |
| No history of TIA, n (%) | 17 (100) | 13 (93) |
| No Diabetes, n (%) | 15 (88) | 13 (93) |
| Smoking, n (%)  No  Ex-smoker  Current smoker | 10 (59)  6 (35)  1 (6) | 7 (50)  7 (50)  0 |
| No hypercholesterolaemia, n (%) | 14 (82) | 11 (79) |
| No history of IHD, n (%) | 13 (77) | 12 (86) |
| Type of stroke, n (%)  Ischaemic  PICH  No relevant abnormality on scan  Died before scan | 12  3  2  0 | 9  0  5  0 |
